# Butanol production from laccase-pretreated brewer’s spent grain

**DOI:** 10.1186/s13068-019-1383-1

**Published:** 2019-03-05

**Authors:** Simona Giacobbe, Alessandra Piscitelli, Francesca Raganati, Vincenzo Lettera, Giovanni Sannia, Antonio Marzocchella, Cinzia Pezzella

**Affiliations:** 1 Biopox srl, Via Salita Arenella 9, Naples, Italy; 2 Dipartimento di Scienze chimiche, Università degli Studi di Napoli“Federico II”, Via Cintia 4, 80126 Naples, Italy; 3 Dipartimento di Ingegneria Chimica, dei Materiali e della Produzione Industriale, Università degli Studi di Napoli “Federico II”, P.le V. Tecchio 80, 80125 Naples, Italy

**Keywords:** Laccase pretreatment, Brewer’s spent grains (BSG), ABE fermentation, Inhibitory compounds, Biobutanol

## Abstract

**Background:**

Beer is the most popular alcoholic beverage worldwide. In the manufacture of beer, various by-products and residues are generated, and the most abundant (85% of total by-products) are spent grains. Thanks to its high (hemi)cellulose content (about 50% w/w dry weight), this secondary raw material is attractive for the production of second-generation biofuels as butanol through fermentation processes.

**Results:**

This study reports the ability of two laccase preparations from *Pleurotus ostreatus* to delignify and detoxify milled brewer’s spent grains (BSG). Up to 94% of phenols reduction was achieved. Moreover, thanks to the mild conditions of enzymatic pretreatment, the formation of other inhibitory compounds was avoided allowing to apply the sequential enzymatic pretreatment and hydrolysis process (no filtration and washing steps between the two phases). As expected, the high detoxification and delignification yields achieved by laccase pretreatment resulted in great saccharification. As a fact, no loss of carbohydrates was observed thanks to the novel sequential strategy, and thus the totality of polysaccharides was hydrolysed into fermentable sugars. The enzymatic hydrolysate was fermented to acetone-butanol-ethanol (ABE) by *Clostridium acetobutilycum* obtaining about 12.6 g/L ABE and 7.83 g/L butanol within 190 h.

**Conclusions:**

The applied sequential pretreatment and hydrolysis process resulted to be very effective for the milled BSG, allowing reduction of inhibitory compounds and lignin content with a consequent efficient saccharification. *C. acetobutilycum* was able to ferment the BSG hydrolysate with ABE yields similar to those obtained by using synthetic media. The proposed strategy reduces the amount of wastewater and the cost of the overall process. Based on the reported results, the potential production of butanol from the fermentation of BSG hydrolysate can be envisaged.

## Background

In recent years, significant steps towards a biobased economy have been taken in order to reduce the emissions of greenhouse gas (GHG) and the dependence from fossil resources. In this frame, the large amounts of waste/residue biomasses from agrofood industries are a key resource to produce both biobased products and second-generation fuels in order to improve the eco-sustainability of productions.

Beer is the most famous alcoholic beverage worldwide. In 2016, the global beer production amounted to about 1.96 billion hectolitres [1], of which about 400 million hectolitres only in Europe [2]. In the manufacture of beer, various by-products and residues are generated, and the most abundant (85% of total by-products) are brewer’s spent grains (BSG) [3]. As reported by Mussatto et al. [3], about 20 kg of wet BSG is generated for 100 L of beer produced. Although BSG is a lignocellulosic material containing sugars (cellulose and hemicellulose), proteins and minerals, its chemical composition depends on several factors, such as cultivation conditions, harvest time, the variety of the barley used, as well as the conditions used for malting and mashing [4].

BSG has been used for several purposes, for example as nutritional source, and for the production of value-added compounds [4]. Thanks to its high (hemi)cellulose content (about 50% w/w dry weight), this secondary raw material is attractive for the production of second-generation biofuels, such as ethanol and butanol. BSG causes trouble when subjected to enzymatic hydrolysis, due to cellulose crystallinity, porosity and its high lignin content (between 12 and 28% w/w dry matter) [4]. The high content of lignin makes a pretreatment step necessary for the saccharification process. Laccases can improve fermentability of lignocellulosic materials mainly through lignin degradation/modification [5, 6]. Delignification by laccases often requires mediators due to complexity and size of lignocellulose materials.

Many works have reported bioethanol production from BSG [4, 7, 8] but, to the best of our knowledge, there is only one report regarding the use of BSG for butanol production [9]. The authors have reported yields of 33 g/kg BSG of butanol and about 41 g/kg BSG of Acetone-Butanol-Ethanol (ABE) starting from acid-pretreated BSG followed by enzymatic saccharification [9].

The purpose of this study is to evaluate the ability of two laccase preparations from *Pleurotus ostreatus* to delignify and detoxify milled BSG in order to improve both saccharification and butanol production through an ABE fermentation process.

A sequential laccase pretreatment and enzymatic hydrolysis process [6], with or without a laccase mediator, was applied to obtain fermentable sugars used as carbon source by *Clostridium acetobutilycum*.

## Results

### BSG characterization

The composition of milled BSG was evaluated following standard NREL protocols (Table 1).

**Table 1:** BSG composition (dry matter)

	Biomass composition (%)
Glucan	16
Hemicellulose (xylan and arabinan)	19
Starch	5
Lignin	21
Ashes	3
Moisture	5
Total phenolic compounds (g/L)	1

Standard deviation ≤ 1%

As reported in the table below, the BSG used in this work contains approximately 40% (w/w) of structural carbohydrates with similar amount of C6 (16% glucan and 5% starch) and C5 sugars (19% of hemicellulose). The BSG contains also an appreciable content of lignin, as a fact, it represents about 20% of the entire biomass, while ashes represent only the 3%. The amounts of the phenolic compounds were 1 g/L.

### Laccase pretreatment

**Fig. 1:**
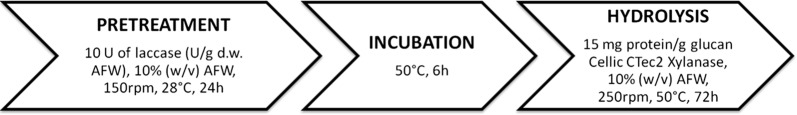
Scheme of sequential protocol

**Fig. 2:**
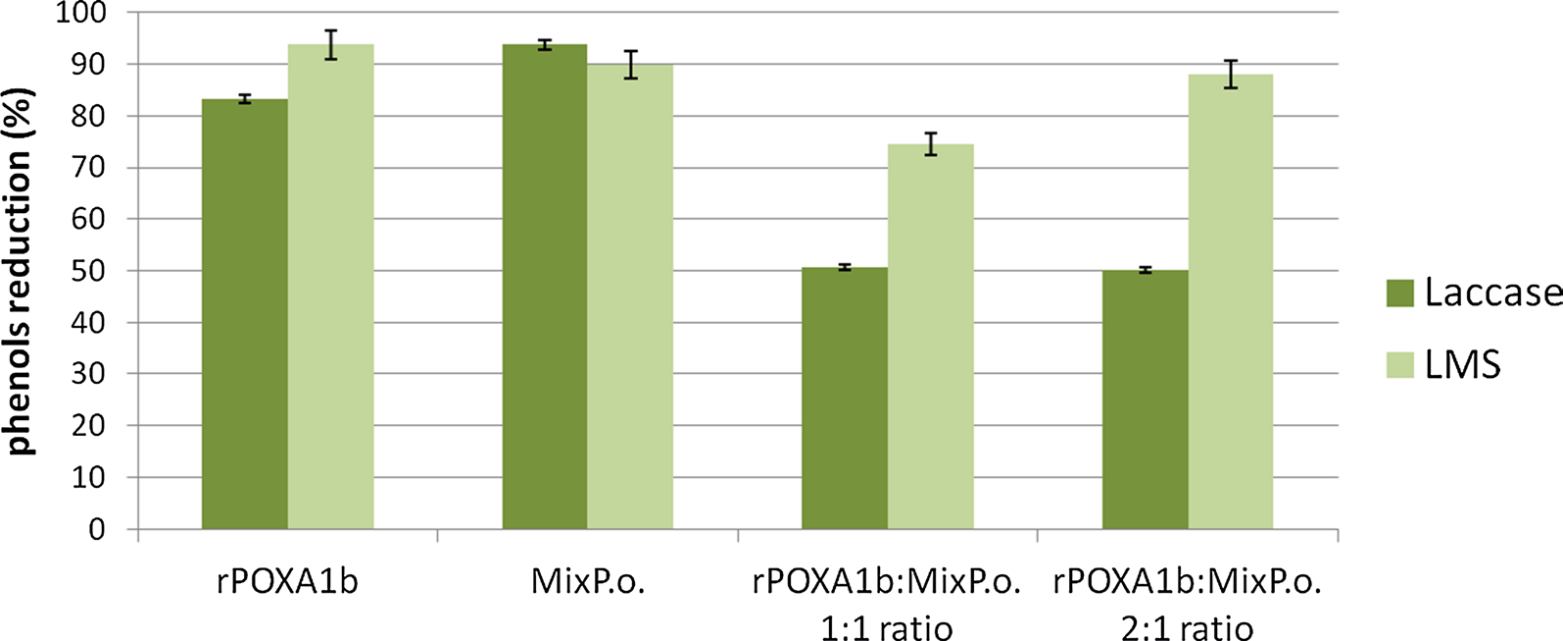
Laccase detoxification. Reduction of phenolic compounds after 24-h enzymatic pretreatment

Sequential enzymatic pretreatment and hydrolysis process was performed as previously described (Fig. 1) by Giacobbe et al. [6]. First of all, the ability of laccase enzyme preparations and of the laccase mediator system (LMS) to reduce the phenol content of BSG was evaluated (Fig. 2). The Mix_P.o_. (rPOXA1b:Mix_P.o._ 0:1 ratio) showed the highest reduction of phenols (94%) followed by rPoxA1b (rPOXA1b:Mix_P.o._ 1:0 ratio) (86%). The LMS gave positive effect in case of rPoxA1b: Mix_P.o._ 1:1 and 2:1 ratios. After laccase pretreatment, evaluation of inhibitory compounds and Klason lignin (KL) content was also performed. As reported in Table 2, the lignin content of the solid residues treated with only rPoxA1b was lower than that of the control (29% delignification) while, when treated with Mix_P.o._, the lignin content was slightly increased, in particular when LMS is used (− 15% and − 43% for laccase and laccase mediator system, respectively). In all other tested conditions, the utilization of the mediator allowed to achieve good delignification yields. No significant variation in other inhibitory compounds content was observed due to laccase action.

**Table 2:** Analysis of KL content and inhibitors in pretreated BSG

rPoxA1b:Mix_P.o._ ratio	KL reduction vs control (%)	Acetic acid (g L^−1^)	Formic acid (g L^−1^)	Furfural (g L^−1^)	5-HMF (g L^−1^)
Control		0.86	0.41	n.d.	n.d.
1:0	29	0.80	0.38	n.d.	n.d.
0:1	− *15*	0.86	0.43	n.d.	n.d.
1:1	1	0.88	0.40	n.d.	n.d.
2:1	10	0.80	0.41	n.d.	n.d.
LMS					
1:0	20	0.83	0.40	n.d.	n.d.
0:1	− *43*	0.85	0.41	n.d.	n.d.
1:1	23	0.85	0.38	n.d.	n.d.
2:1	22	0.86	0.38	n.d.	n.d.

*n.d.* not detected standard deviation ≤ 0.5%. Values indicating lignin increase are highlighted in italic

### Enzymatic saccharification

**Fig. 3:**
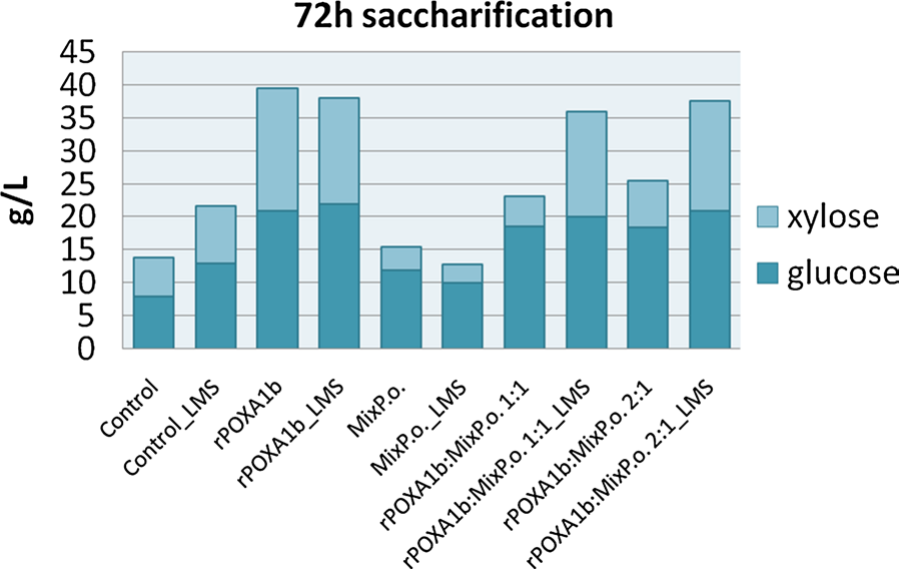
Saccharification results. Sugar yield (g/L) after 72 h of hydrolysis of BSG pretreated with laccases in comparison with control. Standard deviation < 3%

According to BSG composition, the enzymatic cocktail for saccharification was designed including cellulases, xylanase and amylase. The enzymatic saccharification on laccase-pretreated and untreated BSG increased with time, and the best yield was obtained after 72-h hydrolysis (Table 3, Fig. 3). As expected, saccharification yield of untreated BSG was low, yielding up to about 8 g/L of glucose (Fig. 3). When only rPoxA1b was used for the pretreatment, the maximum sugar yield was achieved. As a fact, about 99% of sugar conversion was obtained after 72 h of hydrolysis with a maximum sugars concentration of about 40 g/L, with or without the presence of vanillin as mediator. As expected by results on lignin content, lower sugar yield was achieved when only Mix_P.o._ was used, even when LMS was applied. Analysing saccharification yields of BSG pretreated with the two laccase mixes (rPOXA1b:Mix_P.o._ 1:1 and 2:1 ratios), it is possible to note that the mediator is crucial to increase the sugar yields up to 99%. Based on these results, the hydrolysate from BSG pretreated with rPOXA1b was selected for fermentation tests thanks to the achievement of a sugar concentration able to sustain *C. acetobutylicum* grow, with a balance between C5 and C6 (Fig. 3).

**Table 3:** Saccharification yields

rPOXA1b:Mix_P.o._ ratio used for the pretreatment	Saccharification yields (%)					
	24 h		48 h		72 h	
	No mediator	LMS	No mediator	LMS	No mediator	LMS
Control	1	11	22	36	37	48
1:0	3	17	51	27	99	99
0:1	3	8	20	11	39	29
1:1	4	14	32	36	51	99
2:1	3	15	34	36	75	99

Standard deviation < 3%

### ABE Fermentation

**Fig. 4:**
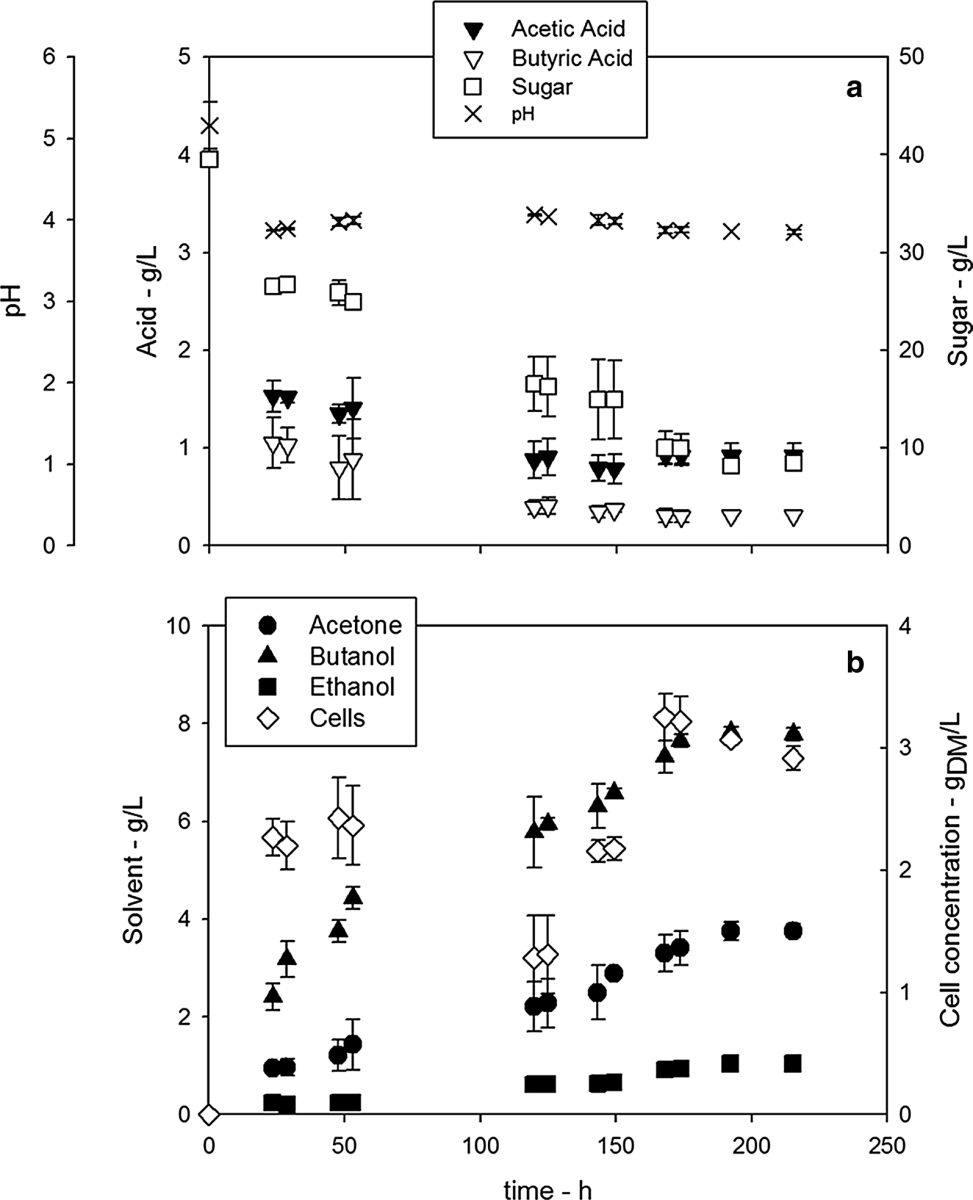
*C. acetobutylicum* fermentation in BSG hydrolysate. Data measured during *C. acetobutylicum* fermentation

*C. acetobutylicum* was able to grow using the BSG hydrolysate as carbon source converting it into ABE. The initial concentration of sugar was about 40 g/L (50% glucose and 50% xylose/arabinose). The pH and the concentration of *C. acetobutylicum* cells, total sugar and metabolites (acetic acid, butyric acid, ethanol, acetone and butanol) as a function of the time are reported in Fig. 4. The analysis of the data confirmed the typical two-phase behaviour of the ABE fermentation [10], such as an initial growth phase (acidogenesis) determining a pH decrease triggering the solvent production phase (solventogenesis). Table 4 summarizes main data regarding the fermentation test carried out with the BSG hydrolysate.

**Table 4:** Relevant data of *C. acetobutylicum* fermentation in BSG hydrolysate

	S_0_ g/L	ξ_S_ %	Residual acids g/L	Y_B/S_ g/g	Y_ABE/S_ g/g	B_MAX_ g/L	ABE_MAX_ g/L
BSG hydrolysate	40±1	78.6±2.0	1.2±0.1	0.25±0.01	0.41±0.02	7.8±0.09	12.6±0.2

S, Sugar concentration; ξ_S_, overall sugar conversion; Y_i/S_, sugar-to-“i-species” fractional yield coefficient; B_MAX_/ABE_MAX_; Maximum fermentation products concentration

In particular, the fermentation test confirmed the high performance typically reported for glucose/xylose fermentation, such as about 79% sugar conversion and ABE production of 12.6 g/L within 190 h. More in detail, 3.8 g/L of acetone, 7.8 g/L of butanol and 1.0 g/L of ethanol was produced. The acidogenesis phase (t_A_) lasted about 1 day. The residual acid concentration at the end of fermentation was 1.2 g/L.

## Discussion

This study reports laccase pretreatment of milled BSG in order to improve both saccharification and ABE fermentation yields. To the best of our knowledge, there is no research work reporting laccase pretreatment of BSG, and only one paper reporting butanol production from this biomass [9].

As known, the composition of BSG depends on several factors, as the variety of barley and harvest time [4]; however, BSG is basically a lignocellulosic material containing carbohydrates, lignin and proteins. Macromolecular composition of the untreated biomass used in this work is similar to that reported by other authors [4, 11]. Analyses indicated that approximately 40% (w/w) of the biomass is composed by structural polysaccharides. This represents a considerable fraction that can be potentially deconstructed into fermentable sugars for the production of biobutanol through ABE fermentation. The amount of lignin in the tested BSG (21% w/w) is relatively high, imposing a pretreatment step to remove this physical barrier against the enzymatic hydrolysis. In particular, the lignin content is similar or slightly lower than that in other lignocellulosic materials pretreated by laccase, such as apple pomace (18%), *Ricinus communis* (19.8%), *Bambusa bambos* (26.5%), coffee silverskin (30%) [6, 11–13]. In our previous work, laccase enzymes from *P. ostreatus* have been successfully used to pretreat milled agrofood wastes with a lignin content ranging from 18 to 33 [6]. In agreement with our previous results, *P. ostreatus* laccases were able to both detoxify and delignify BSG removing up to 94% of phenolic compounds, important inhibitors of microorganisms used in the ABE fermentation. Moreover, the mild conditions of enzymatic pretreatment avoided sugar degradation along with the formation of other inhibitory compounds. The herein obtained detoxification yields are higher than those achieved in our previous work (Table 5) [6]. Delignification results confirmed the tendency of the native mix from *P. ostreatus* to graft phenols, causing increase in the lignin content (− 15% of delignification), with an even more marked effect when mediator is applied (− 43%) (Table 2). In contrast, the main action of rPOXA1b is the lignin degradation, reaching up to 29% of lignin reduction. This different behaviour may be mainly due to the different reactivity of *P. ostreatus* laccases towards phenols generated upon lignin oxidation. It can be envisaged that native mix from *P. ostreatus* further reacted with generated phenols and covalently coupled them back onto the fibres, a phenomenon known as grafting process [14, 15]. Conversely, rPOXA1b did not oxidize these substrates, and therefore no competition between delignification and radical coupling reactions occurred [16].

**Table 5:** Detoxification, delignification and saccharification yields achieved by using *P. ostreatus* laccases on different agrofood wastes

Agrofood waste	Detoxification yield (%)	Delignification yield (%)	Saccharification yield (%)	References
Apple pomace	33	16	83	[5]
Coffee silverskin	69	48	73	[5]
BSG	86	29	99	This work

The delignification results achieved by using rPOXA1b are higher than those reported for apple pomace, a biomass with slightly lower lignin content, but lower than those achieved for coffee silverskin which in contrast contains about 30% of lignin (Table 5).

When compared to other pretreatment methods on BSG, it is possible to observe that delignification yields achieved by laccases are higher than those reported for pretreatments working with harsh conditions [9, 17] and lower than those ones causing also lost of hemicelluloses [17, 18].

It is worth to note that the saccharification yields obtained by Mix_P.o_. are comparable or even lower than those obtained from the untreated BSG, due to the grafting phenomenon ascribable to Mix_P.o_. action, which increases the lignin content, thus hindering hemicelluloses hydrolysis (Table 2, Fig. 2). The presence of rPOXA1b together with Mix_P.o_. increases the delignification and then the saccharification, in particular when the LMS was adopted. As expected, the high detoxification and delignification yields achieved by rPOXA1b resulted in great saccharification. As a fact, no loss of carbohydrates was observed thanks to the novel sequential strategy, and thus the totality of polysaccharides was hydrolysed into fermentable sugars. The BSG hydrolysate, with a concentration of about 40 g/L, contains approximately the same amount of glucose and xylose suitable for *C. acetobutilycum* growth. As a fact, the strain used in this work has been already demonstrated able to grow and ferment both hexose and pentose sugars in solvents (ABE) [19]. When BSG hydrolysate was used as nutrients, the typical behaviour of the ABE fermentation was observed [10]. In particular, the acidogenic phase was characterized by (i) continuous conversion of the substrate; (ii) increase in cell and acid concentration; (iii) pH decrease. As the pH approached 4 (*t*_A_ = 23 h), the solventogenic phase started characterized by (i) gradual decrease in sugar concentration up to a constant value; (ii) steady increase in solvent concentration up to a constant value; (iii) gradual decrease in acid concentration as their conversion by *C. acetobutylicum* is faster than their production. The herein assessed fermentation performance, in terms of both sugar conversion and solvent production, was almost similar to those reported by Raganati et al. [19] on a synthetic medium. The butanol and ABE yields measured in the present investigation, 100 g_B_/kg_BSG_ and 163 g_ABE_/kg_BSG_, are up to 4 times higher than those reported by Plaza et al. [9] using *Clostridium beijerinckii* on BSG at 15% w/w pretreated by sulphuric acid.

## Conclusions

Laccase pretreatment of BSG was successfully carried out by applying the sequential protocol developed in our previous work. *P. ostreatus* rPOXA1b was effective in delignification and detoxification of BSG, allowing hydrolysing the quite totality of carbohydrate in fermentable sugars. *C. acetobutylicum* was applied for conversion of BSG hydrolysate into butanol through ABE fermentation. The fermentation yields were similar to those obtained with synthetic medium and significantly higher than those reported in a previous work from the same biomass. The already developed sequential pretreatment hydrolysis protocol resulted to be effective also for BSG, allowing reduction of the amount of wastewater and the cost of the overall process.

All these results suggest that upon laccase pretreatment, BSG is a promising feedstock to produce butanol.

## Methods

### Brewer’s spent grain

Brewer’s spent grain (BSG) used in this study was kindly supplied by Italian brewery company in the frame of the Waste2fuels project. The supplied biomass was milled (1 < mm > 0.5) and stored under dry conditions at room temperature until further use.

BSG characterization was performed according to Laboratory Analytical Procedures (LAPs) standard protocols of the National Renewable Energy Laboratory (NREL) [20–22].

### Laccase enzymes and activity assay

Two different preparations of laccases from *P. ostreatus* were used in this work: rPOXA1b laccase recombinantly expressed in *Pichia pastoris* [23] and a mix of native laccases (Mix_P.o._) produced by *P. ostreatus* after 10 days of growth in PDY supplemented with 150 μM CuSO_4_ and 2 mM ferulic acid [24]. Laccase activity was assayed as reported by Macellaro et al. [25].

### Laccase pretreatment

The pretreatment was carried out at 10% (w/v) in 50 mM sodium citrate pH 5.0 at 28 °C for 24 h by using 10 U/g of enzymes. The two laccase preparations were used in different combinations: only rPOXA1b (rPoxA1b:Mix_P.o_ ratio 1:0); only Mix_P.o._ (rPoxA1b:Mix_P.o_ ratio 0:1); rPoxA1b:Mix_P.o._ratio 1:1 and 2:1. The effect of LMS was also evaluated by using 2.5% w/v vanillic acid (Sigma-Aldrich) as natural mediator.

Control assays were performed under the same conditions without the addition of laccase. All the experiments were carried out in triplicate.

### Klason lignin evaluation

The Klason lignin content of untreated and pretreated BSG was determined according to NREL LAP protocols [20]. Lignin reduction was estimated as percentage respect to control sample.

### Determination of total phenolic compounds and other inhibitors

Acetic acid, formic acid, furfural, hydroxymethyl-furfural (5-HMF) concentrations were also analysed according to NREL LAPs [21] after the pretreatment. The total phenolic content after pretreatment was analysed by using Folin–Ciocalteu assay [26].

### Determination of protein concentration

Protein concentration was determined with the BioRad Protein Assay (Bio-Rad Laboratories, Segrate (MI), Italy) using bovine serum albumin (BSA) as standard.

### Enzymatic hydrolysis

Enzymatic hydrolysis was carried out directly on pretreated BSG following sequential protocol reported by Giacobbe et al. [6]. The saccharification was performed with commercial Cellic^®^ CTec2 (kindly supplied by Novozyme); endo-1,4-β-Xylanase M1 from *Trichoderma viride* (Megazyme); and α-amylase from *Bacillus licheniformis* (Megazyme) at 50 °C and 250 rpm in a shaking incubator. Sampling was done every 24 h and sugar composition was analysed by following NREL LAP [20, 21].

For all saccharification experiments, 15 mg of enzyme mixture per gram of initial glucan present in BSG was used, as previously described by Giacobbe et al. [27]. Based on BSG carbohydrate composition, the enzymatic cocktail was composed by 80% of Cellic^®^ CTec2 and 10% of xylanase and 10% of amylase. Cellulase complex, Cellic^®^ CTec2, is a blend of cellulases, β-glucosidases and also hemicellulases useful for the degradation of cellulose. This enzyme cocktail was supplemented with an endo-1,4-β-xylanase acting specifically on xylan and also with an α-amylase to break down long-starch chain present in the BSG.

### ABE fermentation

*Clostridium acetobutylicum* DSMZ 792 was supplied by DSMZ. The stock cultures were reactivated according to the DSMZ procedure. The reactivated cultures were stored at − 80 °C. The thawed cultures were inoculated into 12 mL of synthetic medium containing glucose (30 g L^−1^) and yeast extract (YE) (5 g L^−1^) in 15-mL Hungate tubes (pre-cultures). The cells were grown under anaerobic conditions for 48 h at 37 °C, and then they were transferred to fermentation bottles.

The fermentation medium consisted of 5 g L^−1^ YE, 2.5 g L^−1^ NH_4_Cl, 0.25 g L^−1^ KH_2_PO_4_, 0.25 g L^−1^ K_2_HPO_4_ and mineral solution (0.20 g L^−1^ MgSO_4_·7H_2_O, 0.01 g L^−1^ MnSO_4_·H_2_O, 0.01 g L^−1^ FeSO_4_·7H_2_O). The carbon source was the enzymatic hydrolysate of pretreated BSG.

The fermentation tests were performed in 100-mL glass bottles filled with 50 mL of medium, and the pH of the medium was set at 6.5. The bottles were sparged with nitrogen (technical grade) to provide anaerobic conditions. The bottles with the medium were sterilized at 121 °C for 20 min and cooled at room temperature. Before inoculation, 0.5 mL of mineral solution were filter-sterilized (Millipore filter; 0.22 μm) and added to each bottle.

The medium was inoculated with 6.25% (v/v) suspension of active growing pre-cultures. 1 mL of cultures was sampled periodically for cell/metabolite characterization.

The pH was measured off-line by a pH meter (Hanna Instruments). The sugar concentration was determined by high-performance liquid chromatography (HPLC) using an Agilent 1100 system (Palo Alto, CA). The sugars were separated by means of a 8 µm Hi-Plex H, 30 cm 7.7 mm column at room temperature and detected with a refractive index detector. Deionized water was used as mobile phase at flow rate of 0.6 mL min^−1^. The metabolites (acetic acid, butyric acid, acetone, butanol, ethanol) were measured by means of a GC apparatus equipped with a FID and with a capillary column poraplot Q (25 m × 0.32 mm). An internal standard (hexanoic acid) was used to measure acids and alcohols and their concentrations.
